# A case of adrenal infarction in a patient with COVID 19 infection

**DOI:** 10.1259/bjrcr.20200075

**Published:** 2020-06-04

**Authors:** Raekha Kumar, Thushyanthan Guruparan, Safa Siddiqi, Roosey Sheth, Meron Jacyna, Mani Naghibi, Eirini Vrentzou

**Affiliations:** 1 Department of Radiology, Northwick Park Hospital, London North West University Healthcare NHS trust, Watford Road, London HA1 3UJ, UK; 2 Department of Gastroenterology, Northwick Park Hospital, London North West University Healthcare NHS trust, Watford Road, London HA1 3UJ, UK; 3 St Mark's Hospital, Watford Road, London HA1 3UJ, UK; 4 Imperial College, London, UK

## Abstract

This case report highlights an unusual presentation of acute adrenal infarction in a Covid-19 patient who presented with abdominal symptoms and hyponatraemia. We discuss the recent literature reviewing how Covid-19 creates a hypercoaguable state, with acute adrenal infarction as a possible prothrombotic complication.

## Clinical presentation

A 70-year-old female patient with a background history of hypertension and hypercholesterolaemia presented to Northwick Park Hospital with an acute onset of fever, left-sided chest pain, cough and mild dyspnoea. She also reported fatigue, abdominal pain, vomiting and diarrhoea. On examination, she was normotensive and had normal saturations on room air. Her abdomen was mildly distended but not peritonitic. Given the history and current pandemic, she was suspected to be Covid-19 positive.

## Differential diagnosis

There is a wide differential for abdominal pain on a background of Covid-19 infection. Studies have shown that abdominal pain, nausea or vomiting are possible manifestations of Covid-19.^1^ Other potential causes of abdominal pain in this patient included bowel obstruction.

## Investigations

**Figure 1.**
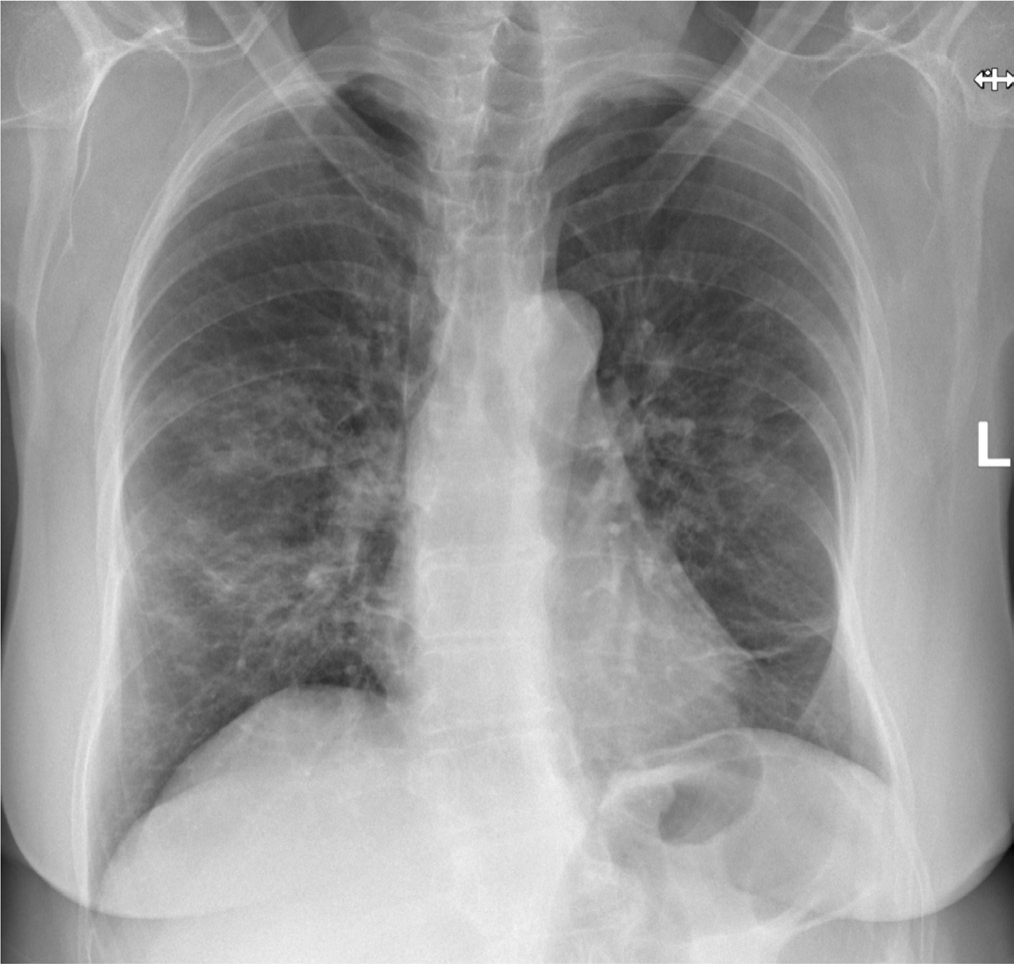
PA chest radiograph demonstrating bilateral, mid and lower zone, patchy parenchymal infiltrates in keeping with moderate Covid-19 pulmonary infection. PA, posteroanterior.

A chest radiograph was performed on admission (Figure 1), which demonstrated patchy airspace consolidation in the mid and lower zones bilaterally, in keeping with Covid-19 infection. This was later confirmed with a positive RT-PCR result from a nasal and throat swab.

**Figure 2.**
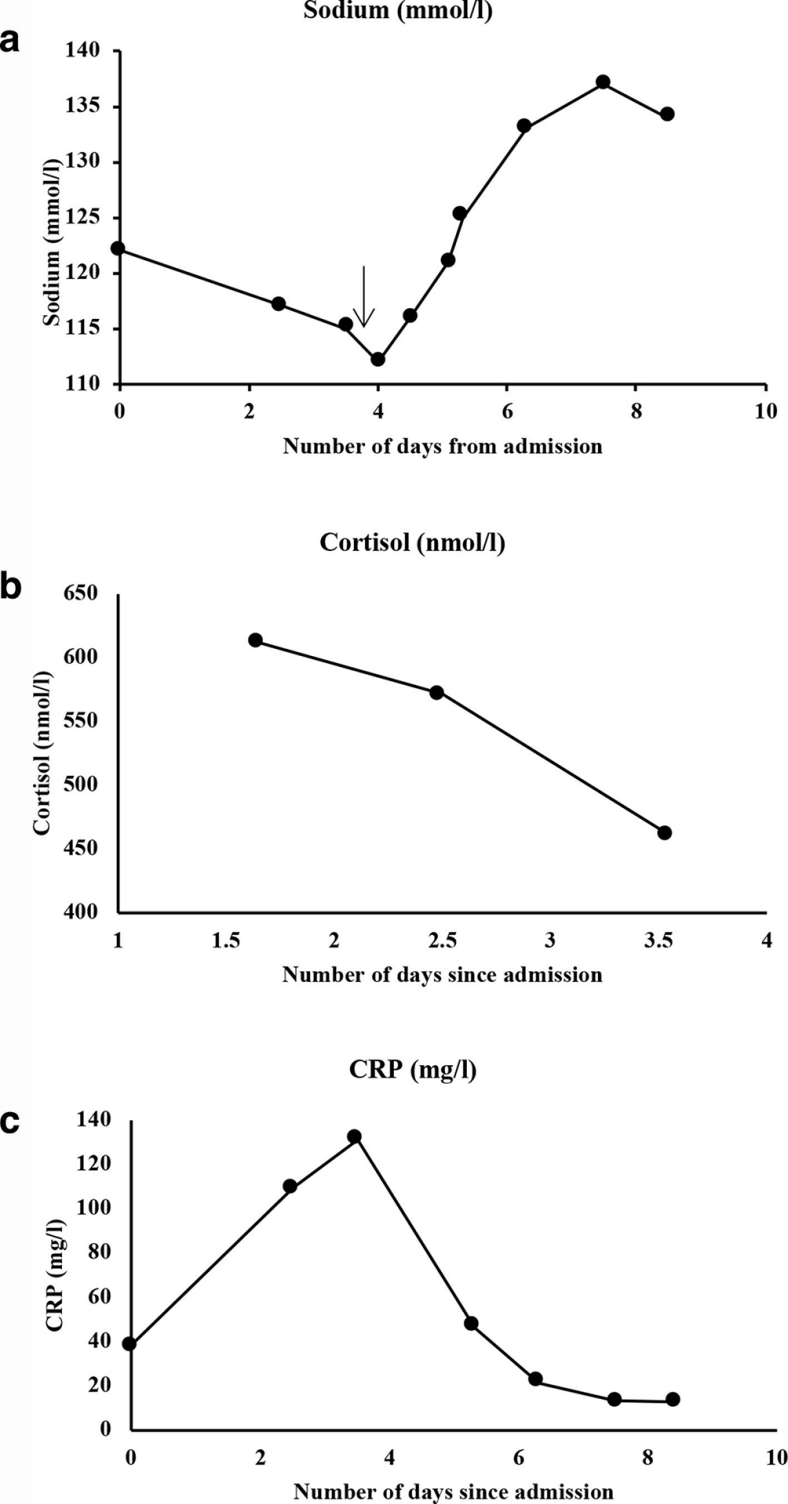
Trends of important blood parameters (sodium, cortisol and CRP) over the duration of the patient’s stay in hospital. The day of treatment commencement for the patient’s hyponatraemia has been demonstrated on the graph 2A (black arrow) with a clear subsequent improvement in the patient’s sodium levels. (Normal ranges: Sodium 133–146 mmol l^−1^, random cortisol expected to be >300 nmol l^−1^ and CRP 1–5 mg l^−1^).

Her routine blood results revealed lymphopaenia of 0.7 × 10^9^/L (normal range: 1.5–4 x 10^9^/L), mildly raised C-reactive protein (CRP) of 38 mg l^−1^ (normal range 0–5mg l^−1^) and normal liver function tests. She had a new hyponatraemia from admission, reaching as low as 112 mmol l^−1^ (normal range: 133–146 mmol l^−1^) and a mildly raised urinary sodium of 46 mmol l^−1^. Her potassium, random plasma cortisol levels and ACTH levels remained within the normal range. Figure 2 demonstrates the trends in sodium, CRP and cortisol. Antibody tests for antiphospholipid syndrome (anticardiolipin antibodies) were also performed at a later stage and were negative.

An abdominal X-ray and a contrast enhanced CT of the abdomen and pelvis were performed on the second day of admission for further assessment of the patient’s abdominal symptoms.

## Imaging findings

The abdominal X-ray showed a mildly distended colon with right-sided faecal loading but no signs of free intraperitoneal air.

**Figure 3.**
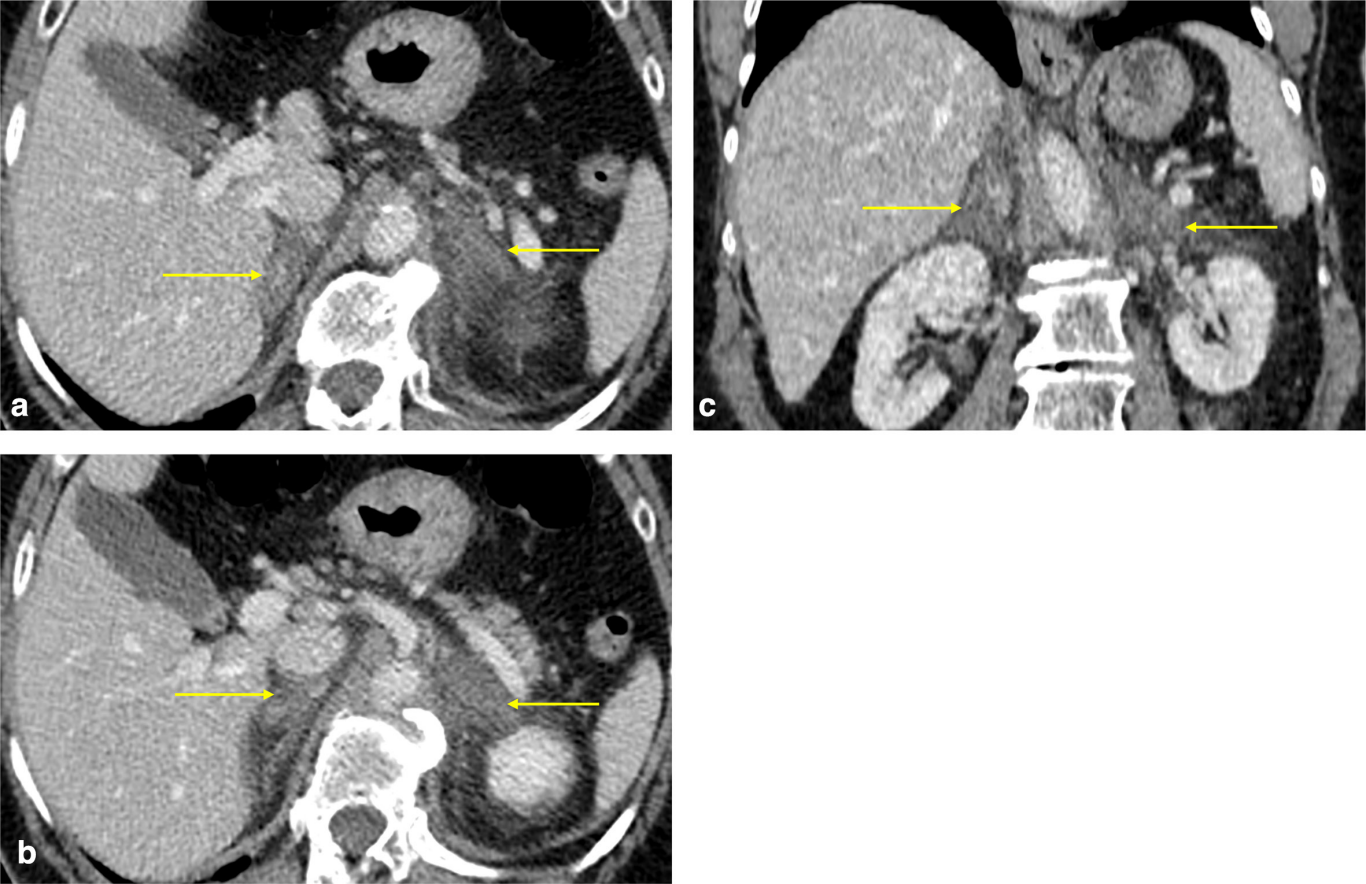
(a, b) Post-contrast axial and (c) coronal CT images of the abdomen on the portal venous phase demonstrating diffusely hypoattenuating, thickened adrenal glands with ill-defined contours and surrounding fat stranding (yellow arrows). Findings are in keeping with acute adrenal infarction.

**Figure 4.**
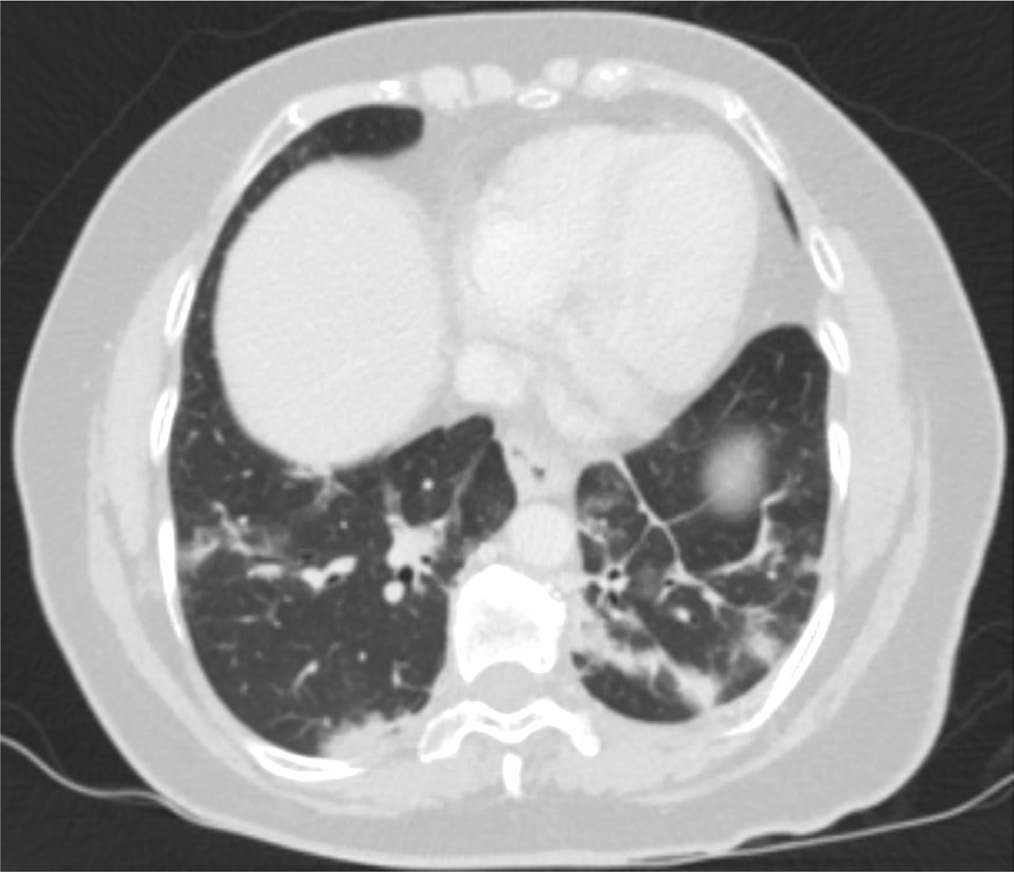
Post-contrast axial images of the CT abdomen and pelvis study at the level of the lung bases (lung window) demonstrating bilateral, peripheral areas of consolidation and ground glass opacities, as well as parenchymal bands. Findings are consistent with Covid-19 pulmonary infection.

A CT study of the abdomen and pelvis with contrast in the portal venous phase identified enlarged, diffusely hypoattenuating adrenal glands demonstrating poor enhancement (**Figure 3**). Their contours appeared ill-defined and there was significant associated surrounding retroperitoneal fat stranding, with no evidence of haemorrhage. This appearance was suggestive of non-haemorrhagic adrenal infarction. No thrombi were visible in the venous or arterial vasculature. The rest of the abdominal viscera were normal. The lung bases included in the study demonstrated bilateral peripheral pulmonary infiltrates with associated ground glass opacities and parenchymal bands in keeping with Covid-19 pulmonary infection (Figure 4).

## Treatment

The patient was managed supportively for Covid-19 and did not require oxygen therapy, in keeping with her relatively mild respiratory symptoms. Her hyponatraemia was thought to be secondary to adrenal insufficiency due to adrenal infarction and the related syndrome of inappropriate secretion of antidiuretic hormone. She was therefore treated with a dose of 200 mg of i.v. hydrocortisone followed by 50 mg of i.v. hydrocortisone 6 hourly. She received oral sodium for 3 days and fluid restriction of 750 mL daily with subsequent rapid improvement in the plasma sodium levels as well as symptomatic relief. The patient was also anticoagulated with warfarin and was discharged on oral hydrocortisone.

## Discussion

The coronavirus 2019 (Covid-19) is a novel RNA betacoronavirus that has been named severe acute respiratory syndrome coronavirus 2.^2^ Common symptoms include fever, cough, fatigue, myalgia and dyspnoea, however there is a significant diversity in patient presentation. A retrospective study in Wuhan demonstrated that in patients with Covid-19 infection, 25% had abdominal pain and 73% had nausea, with other gastrointestinal symptoms including vomiting (65%), diarrhoea (37%) and loss of appetite (98%).^1^

Covid-19 infection is often associated with a marked proinflammatory cytokine response and an elevated CRP in those who are critically ill.^3^ There is a known general association between hyperinflammation and cytokines with the coagulation cascade.^4,5^ In Covid-19, it is hypothesised that increased levels of interleukin-2 and tumour necrosis factor α4 may upregulate coagulation and hence lead to an increased prothrombotic profile with an elevated risk of thrombotic events.^6^ Another potential prothromobotic mechanism is direct damage to endothelial cells by the cytokine response to the virus. The presence of angiotensin converting enzyme (ACE2) receptor, postulated to be used by severe acute respiratory syndrome coronavirus 2 for cell entry on endothelial cells isolated from the vessels of multiple organs, including the lungs and adrenal glands, may be causing direct viral damage to the endothelium in these organs.^7^ Those patients who are severely ill requiring hospitalisation may also have signs of disseminated intravascular coagulation, with an elevated D-Dimer being a common laboratory finding.^8^

There have been several reports highlighting the prothrombotic complications of Covid-19. A Dutch study assessed the incidence of venous thromboembolism and arterial thrombi in ITU patients with Covid-19. They found that despite thromboprophylaxis, there was a 31% incidence of thrombotic complications, with venous thromboembolisms in 27% (pulmonary emboli or deep vein thrombosis) and arterial thrombotic events in 3.7% (all ischaemic strokes).^9^ A thrombotic microangiopathy has also been reported in the pulmonary vasculature which may contribute directly to the ventilation–perfusion mismatch observed in patients alongside the impaired diffusion caused at the alveolar level as a result of the viral pneumonitis, likely contributing to the significant respiratory symptoms noted in Covid-19 and the imaging findings on CT chest studies.^10^ As well as playing a role in the disease process of critically unwell patients, hypercoagulability may also complicate milder respiratory forms of the disease as evidenced by this case of acute adrenal infarction despite relatively mild respiratory symptoms. Transient livedo reticularis, due to skin microthrombi-related venous occlusion, has also been reported in non-critically unwell patients with Covid-19.^11^

To our knowledge, there are no known reported cases of acute adrenal infarction in a Covid-19 positive patient. The adrenal glands are susceptible to infarction and haemorrhage due to their vascular anatomy. They are supplied by multiple arterioles which arise from three different arteries (the inferior phrenic artery, aorta and renal artery), with relatively fewer draining venules connecting to a single suprarenal vein. Prior reports have all suggested that adrenal vein thrombosis secondary to a hypercoagulable state is the initial mechanism leading to adrenal infarction.^12^ It may also be related to microvascular thrombosis in the adrenal parenchyma. This is often complicated by haemorrhage due to secondary adrenal necrosis and/or reperfusion injury or anticoagulant therapy.^13^ Common causes of adrenal infarction include antiphospholipid syndrome (patient tested negative) and pregnancy (usually unilateral), although rarer causes such as heparin induced thrombocytopaenia and myelodysplastic syndrome have been reported.^14,15^ Patients present with symptoms of mineralocorticoid and/or glucocorticoid and/or androgen insufficiency, including fatigue, anorexia, weakness and abdominal pain.

Given Covid-19 has been linked to a hypercoaguable state, we hypothesise that this patient’s acute adrenal infarction was secondary to coronavirus infection. The patient’s main biochemical abnormality was low plasma sodium levels, with normal initial random cortisol and ACTH levels and no hypotensive episodes. The patient’s sodium improved after 3 days of hydrocortisone treatment. This suggests that there was partial adrenal gland infarction with preservation of some function in order to maintain initial cortisol output. We hypothesise that this may be related to microvascular thrombi in the parenchyma, akin to the suggestion of the microvascular pulmonary artery thrombi leading to the pulmonary findings in Covid-19 patients.^9^

This case report highlights a novel finding in a Covid-19 patient which supports the theory that Covid-19 creates a hypercoagulable state with multisystemic implications, including effects on the adrenal glands, and this should be considered even in cases of mild respiratory symptoms. Many Covid-19 patients present with non-specific abdominal symptoms, hence there must be a high index of suspicion to identify adrenal infarction. Clinical assessment and evaluation of laboratory parameters, including sodium, potassium, cortisol and ACTH levels are required along with abdominal CT imaging where appropriate.

## Conclusion

Assessing patients with Covid-19 will remain a challenge during a pandemic that is exerting pressure on our workforce and resources. Clinicians are still gaining knowledge about the multisystemic manifestations of Covid-19 and its prothrombotic effects. We recommend including adrenal infarction in the diagnostic differential of patients who present with abdominal symptoms, as a potential complication of Covid-19 infection.

## Learning points

- 1. Recognition of the wide array of symptoms in Covid-19 positive patients.
- 2. Covid-19 has been related to a prothrombotic state.
- 3. Prothrombotic complications can occur in patients with relatively mild respiratory symptoms.
- 4. A high index of suspicion for adrenal infarction is required in patients with appropriate clinical/laboratory findings.
- 5. Understanding of the CT findings in acute adrenal infarction.
